# Clinical characteristics and peripheral immunocyte subsets alteration of 85 COVID-19 deaths

**DOI:** 10.18632/aging.202819

**Published:** 2021-03-12

**Authors:** Liangkun Xiong, Xin Zang, Guohe Feng, Fangfang Zhao, Shihong Wang, Wenhui Zeng, Kaihuan Yu, Yongzhen Zhai

**Affiliations:** 1 Department of Hepatobiliary Surgery, Renmin Hospital of Wuhan University, Wuhan 430000, China; 2 Department of Infectious Disease, Shengjing Hospital of China Medical University, Shenyang 110004, China; 3 Department of Infectious Disease, Fujian Medical University Affiliated First Quanzhou Hospital, Quanzhou 362000, China; 4 Department of Paediatrics, Renmin Hospital of Wuhan University, Wuhan 430000, China

**Keywords:** COVID-19, SARS-CoV-2, clinical characteristics, lymphocytes, NK cells

## Abstract

Objectives: To retrospectively evaluate the clinical and immunological characteristics of patients who died of COVID-19 and to identify patients at high risk of death at an early stage and reduce their mortality.

Results: Total white blood cell count, neutrophil count and C-reactive protein were significantly higher in patients who died of COVID-19 than those who recovered from it (p < 0.05), but the total lymphocyte count, CD4 + T cells, CD8 + T cells, B cells and natural killer cells were significantly lower when compared in the same groups. Multiple logistic regression analysis showed that increased D-dimer, decreased CD4 + T cells and increased neutrophils were risk factors for mortality. Further multiple COX regression demonstrated that neutrophil ≥ 5.27 × 109/L increased the risk of death in COVID-19 patients after adjustment for age and gender. However, CD4 + T cells ≥ 260/μL appeared to reduce the risk of death.

Conclusion: SARS-CoV-2 infection led to a significant decrease of lymphocytes, and decreased CD4 + T cell count was a risk factor for COVID-19 patients to develop severe disease and death.

Methods: This study included 190 hospitalized COVID-19 patients from January 30, 2020 to March 4, 2020 in Wuhan, China, of whom 85 died and 105 recovered. Two researchers independently collected the clinical and laboratory data from electronic medical records.

## INTRODUCTION

Coronaviruses (CoVs) are a group of viruses that can cause respiratory infections in humans and animals [1]. Since the 21^st^ century, the most serious and concerning coronaviruses are SARS-associated coronavirus (SARS-CoV) and MERS coronavirus (MERS-CoV) which have caused the outbreak of severe acute respiratory syndrome (SARS) in 2002 and Middle East respiratory syndrome (MERS) in 2012 [2, 3]. These two highly pathogenic human coronaviruses mainly invade the lower respiratory tract and cause extrapulmonary manifestations [4]. MERS is not as infectious as SARS, but the fatality rate (34.4%) is higher than SARS (9.5%) [5].

In 2019, a new type of coronavirus named severe acute respiratory syndrome coronavirus 2 (SARS-CoV-2) caused the outbreak of Coronavirus disease 2019 (COVID-19). With the rapid spread of COVID-19 throughout the world, it has constituted a public health emergency of international concern. According to the WHO, the epidemiological curve shows that the pandemic is still developing, the pandemic situation in the northern hemisphere is on the rise, and the number of infected patients in Southeast Asia is increasing rapidly. As of November 17, a total of 53.7 million cases with laboratory-confirmed SARS-CoV-2 infection have been detected and the number of deaths from COVID-19 worldwide has exceeded 1.3 million [6], which means that COVID-19 has become one of the most serious epidemic disasters in human history. With the widespread outbreak of the second wave of COVID-19, reducing patient mortality and improving prognosis have become pressing problems for the world. Most studies in the field of COVID-19 have focused only on the clinical features, but few have analyzed the changes in peripheral immunocyte subsets in patients who died of COVID-19.

In addition to analyzing the clinical characteristics of patients with COVID-19, we also compared the level of immune cells in the recovered patients with the deceased patients, and summarized the changes in various parameters of patients at admission and before death. The impact of these measures on disease prognosis of COVID-19 patients was further analyzed by multivariate logistic regression and multivariate Cox regression. The aim is to identify patients at high risk of death at an early stage and reduce their mortality.

## RESULTS

### Comparison of clinical characteristics between the two groups of patients

As shown in Table 1, 190 cases with laboratory-confirmed SARS-CoV-2 infection were included in this study. Of these patients, 85 patients died during hospitalization and 105 patients were discharged after recovery. The interval between the initial onset of clinical symptoms and hospital admission was 2.0 to 44.0 days (median 10.0 days, IQR 8.0–14.0) in the 85 patients who died in hospital, which was slightly shorter than that of recovered patients (median 13.0 days, IQR 5.8–21.0). Fever and cough were the most common initial clinical symptoms on admission. Less common symptoms included fatigue, chest tightness, shortness of breath, headache, and vomiting. Patients who died were more likely to have fever and shortness of breath than those who recovered (*p* < 0.05). We also analyzed the basic information and clinical characteristics of the 85 deceased patients. The median age of the deceased patients was 71.0 (IQR 65.0–81.0) years, including 25 patients over 80 years of age (29.41%) and 3 patients under 40 years of age (3.53%). The median time from hospital admission to death was 7.0 (IQR 4.0–10.0) days. Seventy (82.35%) deceased patients were diagnosed with at least one chronic disease, and the most common underlying disease was hypertension (54.12%), followed by cardiovascular disease (22.32%), diabetes (21.18%), pulmonary disease (16.47%), cerebrovascular disease (8.24%), kidney disease (8.24%), malignancy (4.71%) and chronic liver disease (2.35%). Respiratory failure was the main cause of death in these patients with COVID-19 (68 patients, 80%), followed by multiple organ dysfunction syndrome (MODS) (11 patients, 12.94%), cardiac arrest (5, 5.88%), and acute coronary syndrome (1, 1.18%).

**Table 1:** Comparison of clinical characteristics between the two groups of patients.

	**Dead cases (*n* = 85)**	**Recovery cases (*n* = 105)**	***P* value**
**Age(years)**	71.00 (65.00,81.00)	58.00 (50.50,69.50)	0.001
>80, *n* (%)	25 (29.41)	11 (10.48)	
40–80, *n* (%)	57 (67.06)	93 (88.57)	
<40, *n* (%)	3 (3.53)	1 (0.95)	
**Sex**			
Male, *n* (%)	53 (62.35)	46 (43.81)	0.011
Female, *n* (%)	32 (37.65)	59 (56.19)	
**Signs and symptoms, *n* (%)**			
Fever	75 (88.24)	78 (74.29)	0.016
Cough	52 (61.18)	62 (59.05)	0.766
Fatigue	22 (25.88)	19 (18.10)	0.194
Shortness of breath	39 (45.88)	26 (24.76)	0.002
Chest tightness	23 (27.06)	19 (18.10)	0.139
Headache	4 (4.71)	1 (0.95)	0.250
Vomiting	2 (2.35)	1 (0.95)	0.833
**Comorbidities, *n* (%)**			
Hypertension	46 (54.12)	29 (27.62)	< 0.001
Diabetes	18 (21.18)	22 (20.95)	0.970
Cardiovascular disease	19 (22.32)	19 (18.10)	0.466
Cerebrovascular disease	7 (8.24)	2 (1.90)	0.089
Kidney disease	7 (8.24)	2 (1.90)	0.089
Lung disease	14 (16.47)	2 (1.90)	< 0.001
Malignant tumors	4 (4.71)	0 (0)	0.082
Chronic liver disease	2 (2.35)	5 (4.76)	0.625
**Cause of death, *n* (%)**			
Respiratory failure	68 (80.00)	0	
MODS	11 (12.94)	0	
Cardiac arrest	5 (5.88)	0	
Acute coronary syndrome	1 (1.18)	0	
Median (IQR) time from onset of symptom to hospital admission	10.00 (8.00,14.00)	13.00 (5.75,21.00)	0.378
Median (IQR) time from hospital admission to outcome	7.00 (4.00,10.00)	17.00 (14.00,27.00)	< 0.001

### Comparison of laboratory findings between the two groups

The laboratory measures were significantly different between patients who died and who recovered from COVID-19. As shown in Table 2, the white blood cell count, neutrophil count and C-reactive protein were significantly higher in the deceased patients than in the recovery patients (*p* < 0.001), while the lymphocyte count and platelet count were significantly lower than the recovered patients (*p* < 0.05). As for coagulation and biochemical indicators, aspartate aminotransferase (AST), total bilirubin, serum creatinine, lactate dehydrogenase, etc. in the deceased patients were significantly higher than in the recovered patients (*p* < 0.05), while no significant difference was observed in the level of alanine aminotransferase (ALT) between the two groups (*p* > 0.05). Albumin concentrations were significantly lower in the deceased group than the recovered group, whereas hypoalbuminemia (albumin < 32 g/L) was more frequent in the deceased group. And compared with the recovered patients, CD4 + T cells, CD8 + T cells, B cells and NK cells of the deceased patients were significantly lower (*p* < 0.05). The CD4 + /CD8 + ratio was not significantly different between these groups (*p* = 0.390).

**Table 2:** Comparison of laboratory indicators between the two groups on admission.

	**Dead cases (*n* = 85)**	**Recovery cases (*n* = 105)**	***P* value**
White blood cells (×10^9^/L)	8.12 (5.54, 12.43)	6.04 (4.68, 7.19)	< 0.001
Neutrophils (×10^9^/L)	6.71 (4.46, 11.21)	3.44 (2.73, 4.85)	< 0.001
Lymphocyts (×10^9^/L)	0.57 (0.38, 0.86)	1.43 (1.11, 2.01)	< 0.001
Platelets (×10^9^/L)	171.00 (115.00, 222.00)	215 (174.75, 272.00)	< 0.001
C-reactive protein (mg/L)	86.70 (45.65,166.55)	5.55 (5.00, 49.50)	< 0.001
Red blood cells (×10^12^ /L)	4.07 (3.65, 4.50)	4.02 (3.77, 4.31)	0.638
Hemoglobin (g/dL)	12.90 (11.10, 13.90)	12.30 (11.70, 13.30)	0.874
Procalcitonin (ug/L)	172.00 (119.50, 226.00)	216.00 (175.00, 275.00)	0.180
D-dimer (μg/mL)	4.65 (1.45, 18.56)	0.63 (0.31, 1.19)	< 0.001
Alanine aminotransferase (U/L)	24.50 (19.00, 44.25)	23.00 (17.00, 41.00)	0.472
Aspartate aminotransferase (U/L)	41.00 (26.25, 61.75)	25.00 (18.00, 31.50)	< 0.001
Alkaline phosphatase (U/L)	65.50 (52.00, 97.00)	70.00 (56.50, 94.50)	0.256
γ-glutamyl transpeptidase (U/L)	34.50 (20.25, 64.75)	28.00 (15.00, 49.00)	0.408
Total protein (g/L)	58.75 (55.13, 63.08)	58.80 (55.70, 62.38)	0.909
Albumin (g/L)	33.30 (31.08, 36.58)	36.90 (34.65, 39.65)	< 0.001
Total bilirubin (μmol/L)	14.10 (9.73, 18.98)	9.90 (7.30, 11.98)	0.026
Urea (mmol/L)	8.42 (5.40, 13.75)	4.76 (3.77, 5.47)	< 0.001
Serum creatinine (μmol/L)	70.00 (53.00, 97.75)	58.00 (49.00, 68.00)	0.017
Creatine kinase (U/L)	85.00 (44.00, 241.00)	55.00 (37.00, 98.50)	0.002
Lactate dehydrogenase (U/L)	478.00 (353.00, 643.00)	220.00 (183.00, 286.00)	< 0.001
CD3 + T cell counts (cells/uL)	297.00 (187.50, 423.00)	966.50 (796.50, 1491.75)	< 0.001
CD4 + T cell counts (cells/uL)	160.00 (99.50, 273.50)	597.50 (389.75, 808.00)	< 0.001
CD8 + T cell counts (cells/uL)	74.00 (48.00, 145.50)	315.00 (218.25, 436.00)	< 0.001
CD4 + /CD8 + ratio	1.79 (1.15,2.94)	1.68 (1.21,3.14)	0.390
CD19 + B cell counts (cells/uL)	92.00 (44.00, 148.50)	244.00 (122.75, 348.25)	< 0.001
NK cell counts (cells/uL)	81.00 (40.00, 158.50)	151.00 (93.25, 233.00)	< 0.001

### Comparison of laboratory parameters of the deceased patients at admission and before death

We compared the laboratory parameters of 65 deceased patients at admission and before death. As shown in Table 3, patients had higher lymphocyte count, albumin and NK cells at admission than before death (*p* < 0.05). However, white blood cell count, neutrophil count, C-reactive protein and D-dimer, etc. increased to varying degrees with the aggravation of patient's condition (*p* < 0.05).

**Table 3:** Comparison of laboratory indicators at admission and before death.

	**On admission**	**Before death**	***P* value**
White blood cells (×10^9^/L)	8.12 (5.53, 12.34)	11.86 (8.01, 17.57)	< 0.001
Neutrophils (×10^9^/L)	6.62 (4.30, 11.16)	11.07 (7.19, 16.38)	< 0.001
Lymphocytes (×10^9^/L)	0.60 (0.41, 0.88)	0.49 (0.34, 0.76)	0.009
C-reactive protein (mg/L)	86.10 (47.00, 166)	103.10 (52.25, 182.55)	0.124
Procalcitonin (ug/L)	0.23 (0.12, 0.71)	1.03 (0.25, 2.26)	< 0.001
D-dimer (μg/mL)	4.56 (1.32, 13.42)	15.26 (4.37, 33.15)	0.007
Alanine aminotransferase (U/L)	24.00 (19.00, 37.25)	36.00 (22.00, 94.00)	0.001
Aspartate aminotransferase (U/L)	39.00 (25.00, 52.00)	47.00 (29.00, 111.00)	< 0.001
Alkaline phosphatase (U/L)	63.00 (51.00, 83.25)	101 (69.00, 130.50)	< 0.001
γ-glutamyl transpeptidase (U/L)	34.00 (21.00, 62.25)	56.00 (31.00, 96.50)	0.014
Total protein (g/L)	58.35 (55.48, 62.7)	58.8 (54.05, 66.35)	0.363
Albumin (g/L)	33.25 (31.60, 36.52)	29.95 (27.07, 32.73)	< 0.001
Total bilirubin (μmol/L)	14.60 (10.38, 18.52)	21.05 (12.47, 29.43)	0.003
Urea (mmol/L)	8.37 (5.40, 13.22)	12.50 (8.77, 26.37)	< 0.001
Serum creatinine (μmol/L)	70.00 (53.00, 97.25)	78.00 (55.50, 195.00)	0.030
Creatine kinase (U/L)	82.00 (45.00, 173.50)	165.50 (62.75, 610.00)	< 0.001
Lactate dehydrogenase (U/L)	457.00 (348.00, 589.00)	683.00 (487.25, 896.25)	< 0.001
CD3 + T cell counts (cells/uL)	297.00 (191.50, 471.50)	217.00 (136.00, 287.00)	0.279
CD4 + T cell counts (cells/uL)	181.50 (106.25, 280.50)	143 (97.00, 199.00)	0.360
CD8 + T cell counts (cells/uL)	90.50 (52.00, 152.25)	55.00 (36.00, 88.00)	0.433
CD4 + /CD8 + ratio	1.70 (1.09,2.90)	2.34 (1.15,3.43)	0.648
CD19 + B cell counts (cells/uL)	93.50 (52.00,143.50)	85.00 (53.00,178.00)	0.522
NK cell counts (cells/uL)	86.00 (51.00,159.25)	48 (25.00,91.00)	0.002

### Risk factors of mortality in COVID-19 patients

To analyze the association of clinical and laboratory parameters with the risk of death, we performed a multiple logistic regression analysis. Table 4 showed that increased D-dimer, decreased CD4 + T cells, and increased neutrophils were risk factors for death. D-dimer (OR = 1.190, 95%CI 1.025–1.382, *p* = 0.022) and neutrophils (OR = 1.709, 95%CI 1.021–2.863, *p* = 0.042) were positively associated with the risk of in-hospital mortality. CD4 + T cells (OR=0.982, 95%CI 0.971–0.993, *p* = 0.001) were inversely associated with the risk of death.

**Table 4:** Multivariate Logistic regression analysis for mortality risk factors.

**Variables**	**OR (95% CI)**	***P* value**
Age	1.079 (0.958–1.215)	0.212
Gender	0.833 (0.051–13.687)	0.898
D-dimer	1.190 (1.025–1.382)	0.022
CD4 + T cell counts	0.982 (0.971–0.993)	0.001
Neutrophil counts	1.709 (1.021–2.863)	0.042

Abbreviations: OR = odds ratio, CI = confidence interval.

**Figure 1:**
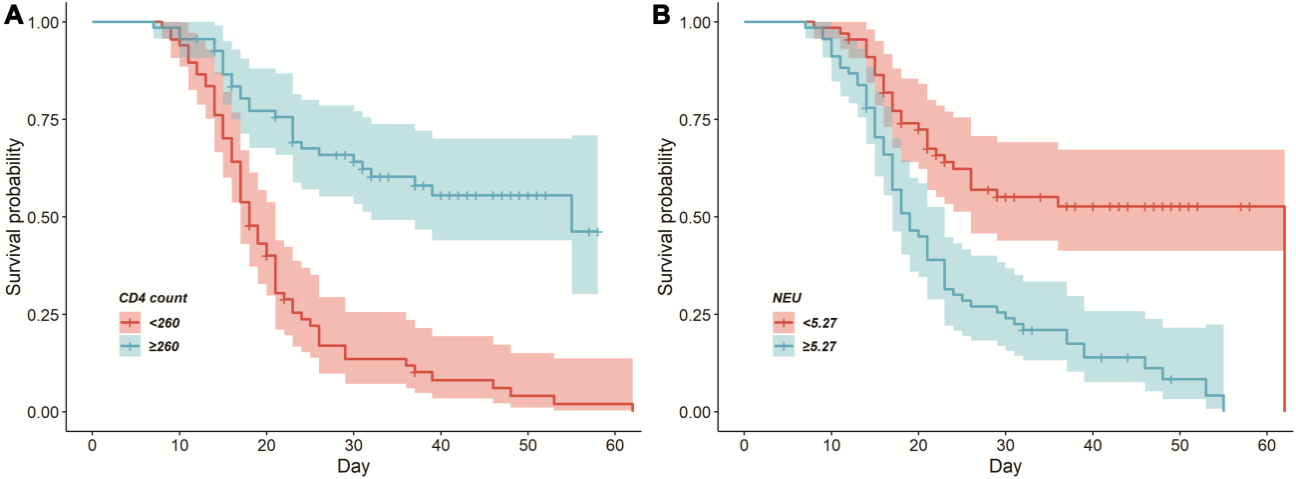
**Cumulative survival curves among the deceased patients with COVID-19.** (**A**) CD4 + T cells ≥260/μL and CD4 + T cells <260/μL on admission. (B) Neutrophil ≥5.27 × 10^9^/L and neutrophil <5.27 × 10^9^/L on admission.

Further survival analysis revealed that CD4 + T cell counts and neutrophil counts remained significant for the prediction of mortality risk in COVID-19 patients. We took the median of the laboratory parameters of all patients for group analysis and plotted a Kaplan-Meier (K-M) survival curve (Figure 1). As shown in Table 5, after adjustment for age and gender, neutrophils ≥5.27 × 10^9^/L (HR = 2.010, 95%CI 1.256–3.217; *p* = 0.004) may increase the risk of death. However, CD4 + T cells ≥ 260/μL (HR = 0.420, 95%CI 0.256–0.689; *p* = 0.001) appeared to reduce the risk of death.

**Table 5:** Multivariate COX regression analysis for the prognosis of COVID-19.

**Variables**	**HR (95% CI)**	***P* value**
Age	1.000 (0.984–1.017)	0.986
Gender	0.697 (0.443–1.097)	0.119
CD4 + T cell counts≥260/μL	0.420 (0.256–0.689)	0.001
Neutrophil counts≥5.27 × 10^9^/L	2.010 (1.256–3.217)	0.004

Abbreviations: HR = hazard ratio, CI = confidence interval.

## DISCUSSION

The immune system plays an important role in maintaining the stability of the internal environment. SARS-CoV-2 infection caused imbalance in the level of lymphocyte subsets, leading to immunologic dissonance. In our study, we observed that SARS-CoV-2 infection caused a significant decrease in circulating lymphocytes. Comparing the immune function of the deceased patients with the recovered patients with COVID-19, we found that CD4 + T cells, CD8 + T cells, B cells and NK cells deceased more severely in the deceased patients. T cells play an important role in the immune response against viral infections. CD4 + T cells can promote the proliferation and differentiation of B lymphocytes and assist B cells to produce virus specific antibodies [7, 8]. A recent study showed that infection and exposure to SARS-CoV-2 induced persistent memory T cells, which may be helpful in the development of a vaccine against COVID-19 [9] Activated CD8 + T cells, also called cytotoxic T cells (CTL), act by producing cytokines TNF-α and IFN-γ [10], or by releasing cytotoxic molecules such as perforin and granzyme to kill target cells [11]. SARS-CoV-2 triggers the release of cytokines, excessive cytokines cause a significant decrease in T cell counts, and surviving T cells also experience functional failure [12], making patients more susceptible to secondary infections. Previous studies have showed lymphopenia was associated with poor prognosis in patients with COVID-19 [13, 14], which is consistent with the findings in our study. By multivariate logistic regression, our results suggested that the decrease in CD4 + T cells was a risk factor for death in COVID-19 patients. Further multivariable Cox regression model revealed that CD4 + T cells ≥ 260/μL appeared to reduce the risk of death. Natural killer (NK) cells are an important component of the innate immune system and are associated with virus clearance and immune regulation [15]. Activated NK cells lead to cytotoxic degranulation and the production of inflammatory cytokines, thereby achieving the purpose of killing target cells [16]. In our study, we found that NK cell counts of the deceased patients were significantly lower before death than at the time of admission [17]. Although innate immunity is activated before specific immunity, our results showed a gradual decline in innate immunity after onset. A recent study found that severe patients not only experienced decreased lymphocyte counts, but also slowed down immune cell response in the innate immune system, resulting in suppressed innate immune function [18]. For clinicians, dynamic monitoring of human immune function indicators may be helpful in assessing the severity of the disease. In the field of cell therapy, *in vitro* experiments have verified that NK cells are capable of inhibiting and eliminating various viruses such as SARS-CoV and acquired immunodeficiency syndrome virus (HIV) [19], and NK cell therapy experiments for COVID-19 have been initiated.

In terms of laboratory findings, leukocytosis (WBC≥10 × 10^9^/L) were more common in the deceased patients than in the recovered patients, which may be related to the higher rate of bacterial infection in those critically ill patients. Elderly COVID-19 patients with underlying chronic diseases are more likely to develop leukocytosis which may lead to critical consequences [20]. In addition, we found that an increase in neutrophil count was strongly associated with a poor prognosis in patients with COVID-19, and the neutrophil count continued to rise until death in most patients who died. Neutrophils are the first innate immune cells that penetrate the site of infection to eliminate invading pathogens [21]. In addition to killing pathogens directly, neutrophils can also produce cytokines to enhance the host immune response [22]. However, excessive accumulation of neutrophils can cause excessive inflammation, cytokine storm and tissue damage, and even increase the severity of the disease, which has been observed in patients infected with influenza A virus [21, 23].

Multiple studies have confirmed that elevated D-dimer is a risk factor for death in patients with COVID-19 [24, 25], which is consistent with our study. D-dimer is a fibrin degradation product that reflects the severity of hypercoagulable state [26]. COVID-19 lesions are primarily in the lungs, but often involve multiple organs [27]. Previous studies found liver dysfunction occurred in 14–53% of patients with COVID-19, and liver damage was more common in severe patients than mild patients [28]. A study on liver injury supports the concept that an older age predispose to more severe liver damage from COVID-19 [29]. More intensive monitor of liver function and individually tailored therapeutic approaches are needed for elderly patients, especially among those with liver disease [30]. Several reports have shown that the binding of the spike protein to the human receptor protein ACE2 is the first step for the SARS-CoV-2 virus to infect the body [31, 32]. In liver, most of the bile duct cell clusters (59.7%) expressed abundant ACE2 cell surface receptors, which were significantly higher than those of hepatocytes (2.6%) [33]. Alkaline phosphatase and γ-glutamyl transpeptidase are enzymes related to bile duct cells, and there was no significant difference in ALP and γ-GGT between the deceased group and the recovered group in our study. These results indicated that the liver damage may not be caused by the virus acting on the bile duct cells. Meanwhile, we found that the deceased patients had significantly higher AST level on admission than the recovered patients. However, there was no significant difference in ALT level between the two groups. With the aggravation of the condition in those who died of COVID-19, the levels of ALT and AST were significantly higher before death than at admission. Liver biopsy of the deceased patients showed mild lobular and portal vein activity and moderate microvascular steatosis [11]. Previous studies have demonstrated that a cytokine storm is triggered when the immune system overreacts to infection [34, 35]. And cytokine storm is considered to be an important factor leading to acute respiratory distress syndrome and multiple organ dysfunction syndrome [36]. Therefore, we speculate that liver damage may be caused by excessive immune response or multiple organ dysfunction at the end of the disease. The mechanism of SARS-CoV-2 induced liver damage may need to be further explored.

This study also has several limitations. Wuhan is the city with the earliest outbreak of COVID-19 in the world. Due to the limitations of early recognition of the disease, patients admitted early often lack laboratory data, such as certain cytokines (IL-6, IL-10, TNF-α, IFN-γ). In addition, whether the results of this study can provide an early prediction and assessment of the risk of patients with severe COVID-19 needs to be further verified in clinical diagnosis and treatment.

## MATERIALS AND METHODS

### Study design and participants

We retrospectively collected the clinical characteristics of COVID-19 patients who were laboratory-confirmed in the East Campus of the Renmin Hospital of Wuhan University from January 30, 2020 to March 4, 2020. The East Campus of the Renmin Hospital of Wuhan University, the third batch of medical institutions for COVID-19 designated by the National Health Commission, is responsible for the treatment of severe and critically ill patients with COVID-19. This study protocol meets the requirements of the Medical Ethics Committee of Renmin Hospital of Wuhan University. Written informed consent was waived due to the urgency of the COVID-19 pandemic. We retrospectively analyzed the clinical data and did not involve potential risks.

### Data collection

According to the guidelines for the diagnosis and treatment of SARS-CoV-2 issued by the Chinese National Health Committee (the 4^th^ edition), we used real-time reverse transcriptase polymerase chain reaction (RT-PCR) to detect nasopharyngeal swabs, and all patients participating in this study were defined as having positive results. Patients under the age of 18, pregnant and lactating women, and patients transferred to other designated hospitals during hospitalization were excluded. Therefore, 190 patients were included in the final analysis. We extracted the basic information of patients from the electronic medical records of the hospital which include clinical symptoms, signs, comorbidities, laboratory test results, etc. Laboratory tests included complete blood cell count, liver function, kidney function, immune function, blood coagulation function, etc. Two researchers independently reviewed the data collection form to verify the accuracy of the data.

### Statistical analysis

Continuous variables were presented with mean and standard deviation or median and interquartile range (IQR), and count data was described by the number of cases and percentage (%). First, univariate analysis was performed on the influencing factors of the deceased group and the recovered group. Non-parametric tests were used for continuous data, and χ² test, chi-square correction or Fisher's exact test were used for disordered categorical data. Factors that were statistically significant in univariate analysis and those considered to be influential by expertise were then included in the logistic regression analysis. In order to avoid collinearity, we adopted a stepwise regression analysis approach, incorporating age and gender into the model based on our knowledge of COVID-19. The remaining variables were selected according to the changes in AIC. Finally, the model with the smallest AIC was selected as the final influencing factor of death. And *p* < 0.05 was considered to indicate a statistically significant difference. Statistical analysis was performed using R4.2.
